# A Stochastic Model for Electron Multiplication Charge-Coupled Devices – From Theory to Practice

**DOI:** 10.1371/journal.pone.0053671

**Published:** 2013-01-31

**Authors:** Michael Hirsch, Richard J. Wareham, Marisa L. Martin-Fernandez, Michael P. Hobson, Daniel J. Rolfe

**Affiliations:** 1 Central Laser Facility, Research Complex at Harwell, STFC Rutherford Appleton Laboratory, Harwell Oxford, Didcot, United Kingdom; 2 Department of Engineering, University of Cambridge, Cambridge, United Kingdom; 3 Department of Physics, University of Cambridge, Cambridge, United Kingdom; The University of Chicago, United States of America

## Abstract

Electron multiplication charge-coupled devices (EMCCD) are widely used for photon counting experiments and measurements of low intensity light sources, and are extensively employed in biological fluorescence imaging applications. These devices have a complex statistical behaviour that is often not fully considered in the analysis of EMCCD data. Robust and optimal analysis of EMCCD images requires an understanding of their noise properties, in particular to exploit fully the advantages of Bayesian and maximum-likelihood analysis techniques, whose value is increasingly recognised in biological imaging for obtaining robust quantitative measurements from challenging data. To improve our own EMCCD analysis and as an effort to aid that of the wider bioimaging community, we present, explain and discuss a detailed physical model for EMCCD noise properties, giving a likelihood function for image counts in each pixel for a given incident intensity, and we explain how to measure the parameters for this model from various calibration images.

## Introduction

Electron multiplication (EM) charge-coupled devices (CCD) are used to take images under low-light conditions and for photon-counting experiments. They are applied in a wide range of scientific fields, such as single molecule microscopy, astronomy, spectroscopy and biomedical imaging. Imaging under low-light conditions presents the problem that the signal can be low compared to the readout noise. EMCCDs overcome this problem by amplifying the signal in an electron-multiplication register. This reduces the effective readout noise to less than one electron. This comes at the price, however, of introducing an additional source of noise.

Having been pioneered in fields such as astronomy, the importance of both Bayesian and maximum-likelihood methods for obtaining robust and accurate quantitative results from analysis of image data is increasingly being recognised in other fields, in particular bioimaging [1]–[5]. Understanding the significance and accuracy of results depends crucially on a detailed characterisation of the noise properties of the imaging system and Bayesian methods allow optimal exploitation of this knowledge to draw objective conclusions from observations. Therefore, in order to enable robust quantitative analysis of EMCCD image data, we need to understand the noise properties of the imaging process. A convenient form for this noise model is a likelihood function, the probability of measuring a particular image value in a pixel given the value of the incident intensity for that pixel.

Rather than giving an explicit model for the noise, measurement errors can also be estimated numerically, for instance via bootstrapping [6], although this process can be computationally expensive and is still more limited than a full Bayesian approach in that there are little to no opportunities for making use of prior knowledge and belief.

There have been extensive investigations of the noise behaviour of EMCCD cameras, for instance [7]–[11]. These works provide a wide knowledge-base of the noise behaviour of EMCCDs. [12] measured the excess noise of the electron-multiplication register. [7] used the knowledge of the likelihood to estimate the ratio of single photons that can be counted using the cut-off method. [13] also considered EMCCD noise characteristics to assess their performance in the photon-counting regime. Attempts to provide a model for the likelihood function have been made [14], However, this model is not appropriate for an EMCCD. Also [10] and [7] used probability density functions (PDF) to model parts of the EMCCD without taking full advantage of the result. A recently [15] published work used a detailed noise model likelihood for an EMCCD, exploiting it for maximum-likelihood scintillation detection.

Recently further papers have appeared which use or advocate the use of Bayesian approaches to analyse data but many still assume simple noise models, commonly a normal or Poisson distribution (e.g. [2]–[4], [16]) either for computational efficiency or possibly due to lack of awareness of a better model or how to make use of one. In an effort to advance our own data analysis capabilities in the field of single molecule imaging in live cells, we developed and tested a detailed noise model likelihood function for EMCCDs. This work was performed independently of [15] and resulted in the same final model. We will show that empirical properties of the EMCCD noise, such as the excess noise factor can be derived from this model. In contrast to [15] however, in this paper we present and explain this model in detail, test it and explain how to calibrate it, so that the wider biological imaging community can make better use of advanced quantitative data analysis techniques for EMCCD images.

We will first give a short overview of the sources of noise and some systematic contributions. Next we motivate and derive the model for the probability distribution and finally we will suggest methods for estimating the parameters upon which the model depends.

## Results

### Sources of Noise and Bias Subtraction

**Figure 1:**
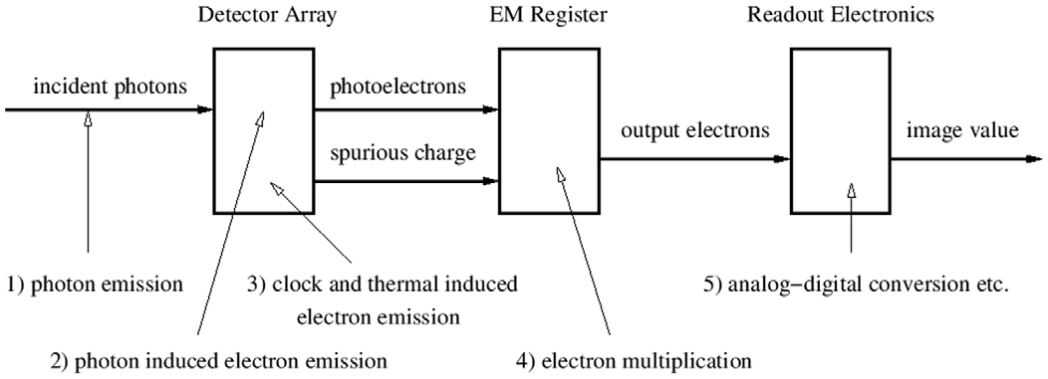
Schematic of the sources of noise during the photon measurement.

**Figure 2:**
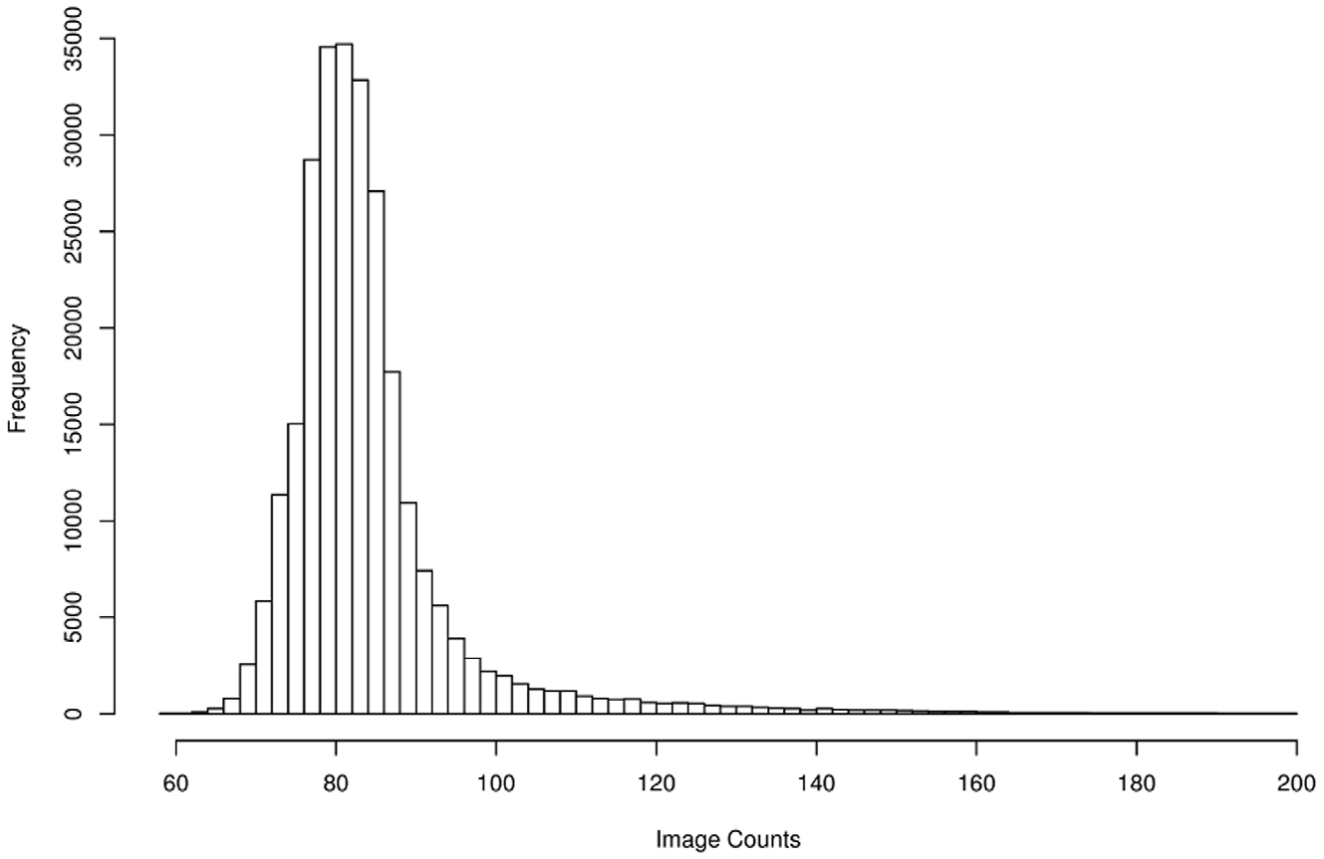
Histogram of the experimentally observed image counts from a dark image. The data was taken with an EM gain of 300.

In order to understand the different sources of noise that affect low-light measurements, we consider the path of the signal through the instrument, see Fig. 1. For more details, see for instance [17]. The first source of noise results from emission of photons from a light source. The detector component of the EMCCD consists of a number of bins (pixels). The bins are combined to form a detector array, which has an exposed and a covered part plus a readout register. When a photon hits the exposed part of the detector array there is a chance that it creates a photoelectron. This stochastic process is the second source of noise. The number of electrons expected per photon is the quantum efficiency of the detector. The third source of noise stems from spurious charge, which consists of two components. The read-out process shifts the electrons through the system of bins by means of changing electrode voltages. During the shift process there is a chance that unwanted electrons are created, which is known as clock induced charge (CIC). The CIC occurs in the detector array and readout register as well as in the EM register. [13] discusses a model for the CIC that includes the EM register. We consider the CIC in the detector array only, since this yields sufficiently accurate results. The CIC depends on the vertical clock speed at which the rows of the detector array are shifted towards the readout register. The detector array is also affected by thermally induced dark current, which is usually reduced by the cooling of the detector. While the dark current is time-dependent, the CIC only depends on the number of readout processes. However, for a particular exposure duration and EMCCD configuration, from the point of view of a noise model, the spurious charges coming from CIC and dark current can be considered to be one source of noise. We assume that the charge transfer efficiency, the fraction of electrons actually transferred from one bin to another, [7] is 100%. In the EM register, the electrons are shifted using a higher clock voltage than in the detector array and readout register to create more electrons through impact ionisation, which is also a stochastic process and the fourth source of noise. Finally, the electronics that amplifies the signal and converts it into discrete image values creates read-out noise. The sole purpose of the EM register is to increase the signal well above the readout noise, so that the effective read-out noise is less than one photon. Figure 2 shows a histogram of a dark image showing the readout noise (variance of the peak at 80 image counts) and the amplified spurious charge (tail).

The A/D converter introduces another source of noise – the quantisation noise – due to the transformation of a continuous value into a discrete value. However, since the A/D factor is moderate, the quantisation can be ignored. The EMCCD models used in this paper have a A/D factor of 10–13 electrons per image value at maximum pre-amplification gain. The pre-amplifier is a part of the readout electronics. That means, for an EM gain factor of 250 we expect 19 to 25 image counts per electron that enters the EM register. Under such circumstances, the quantisation noise is a small fraction of an electron.

**Figure 3:**
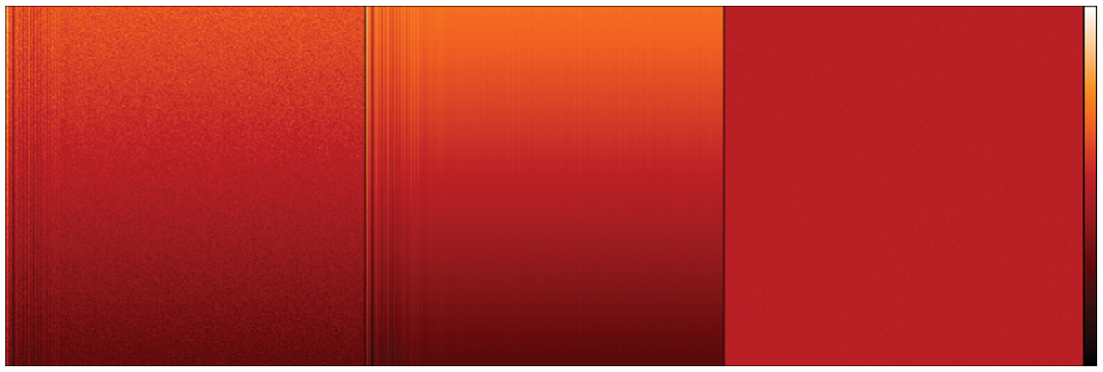
Removal of systematic contributions. Colour coded, experimentally observed image counts from a dark image. The strip on the far right shows the colour code assignment, where black corresponds to the lowest observed image value and white corresponds to the highest observed image value. From left to right: the original, uncorrected dark image; the image of systematic contributions and the dark image after contributions have been subtracted.

Beside the noise that is created during read-out, there are also some systematic contributions from the detector, which we will briefly consider. To that end we have taken dark images, i.e. images taken with the detector array covered, but with the usual exposure time, which therefore only show spurious charge and noise. Dark images are usually not homogeneous, Fig. 3 left. We calculated the row and column mean values. The row means are not purely statistical but have a gradient in intensity. The columns show systematic effects in the form of lines. The particular pattern of these effects changes from detector to detector and is also dependent on the detector settings. Depending on the EM gain setting, the details of these variations across the image may be insignificant. However, the measurement and subtraction of the constant bias offset (added electronically to avoid negative values) to these images is necessary to understanding the noise, and can be achieved easily by the process below which also removes these systematic contributions.

To remove systematic contributions, we determine these contributions from dark images. The contributions to the value (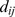) of the pixel in row 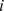 and column 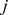 come from noise including spurious charge (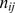) and components that are constant over the whole image (bias offset, 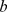), a row (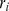) or a column (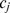), respectively:
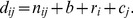
(1)


The mean values for a row, a column and the total image are given by:
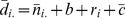
(2)

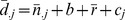
(3)

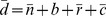
(4)


The dot in the index is a place holder to indicate whether the mean was taken over rows or columns. To remove the systematic contributions from an image composed of the signal and dark image, 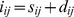, we subtract the column and row means of the dark images for each pixel and add the mean of the total dark image:
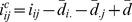
(5)

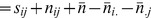
(6)


The transformed image contains only signal and noise components. If the noise is uncorrelated and isotropic, we can assume that 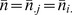 and we get
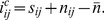
(7)


Spurious events are rare. If we assume that the mean noise value is dominated by Gaussian read-out noise with mean 0, then we get 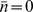 and 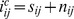. Alternatively, all calculations could also be done using the median instead of the mean. The amplified spurious charge will act as outliers. It will pull the mean to slightly higher values. The median is less affected by such effects. However, the practical difference is negligible.

Both components of the corrected image contain noise. The signal contains the Poisson noise of the light source, the noise from the creation of photoelectrons and the EM noise for the photoelectrons. The “noise” term contains the Poisson noise of the spurious charge modified by the EM noise and the read-out noise. The emission of spurious charge is itself a stochastic process that has an expected value and a variance.

### The Detector Model

Key to the application of Bayesian or maximum-likelihood methods for robust quantitative analysis of CCD images is the likelihood of measuring a particular number of electrons in a CCD pixel for a given input signal. If the expected number of incident photons hitting a pixel is 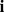 and the measured number of image counts in the pixel is 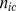, this probability density function is 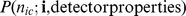. This likelihood function is the noise model for our problem. Given this function and a parametrised model (or models) for the variation of 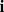 across the CCD, 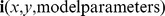, objective determination of model parameters with confidence limits is possible, as is robust model selection and choosing between alternative possible models to explain the data. Increasingly challenging imaging problems, e.g. understanding noisy, crowded images of single molecules in cell membranes, demand such an approach to open up new avenues of research.

We want to know the probability of obtaining 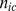 image counts in a pixel if light of a certain intensity hits the detector. To achieve that we combine the five sources of noise step-by-step. The result is a model that is a combination of a Poisson distribution, a gamma distribution and a normal distribution. We will refer to the model as the PGN model.

#### The Poisson contribution

The photons incident on a detector pixel follow a Poisson process [18] with the mean intensity 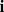. We will denote the parameters of the model with bold lower case letters (for a reference of notation see table 1). The probability that 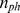 photons hit the detector pixel is therefore given by a Poisson distribution 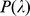 with mean 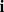:
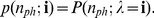
(8)


**Table 1:** Table of mathematical symbols.

model parameters	
**I**	light intensity in photons
**q**	quantum efficiency of the detector in electrons per photon
**c**	spurious charge (dark current and clock induced charge) in electrons
**g**	gain of the electron multiply (EM) register (dimensionless)
**r**	readout noise in electrons
**f**	A/D factor in electrons per image value
Θ	generic parameter for the CCD specifications
quantities of the signal flow	
*n_ph_*	number of photons
*n_pe_*	number of photoelectrons
*n_ie_*	number of input electrons of the EM register
*n_oe_*	number of output electrons of the EM register
*n_ic_*	number of image counts (pixel value of the digital image)
probability distributions and models	
*B*(*n*, *p*)	Binomial distribution of *n* trials with probability *p*
*F_χ_*(*k*, *l*)	non-central chi-square distribution
	with *k* degrees of freedom and non-centrality parameter *l*
*γ*(*k*, *θ*)	gamma distribution with shape parameter *k* and scale parameter *θ*
*G*(*k*, *θ*)	augmented gamma distribution, see eqn. (20)
*N*(*μ*, *σ*)	normal distribution with mean *μ* and standard deviation *σ*
*P*(*λ*)	Poisson distribution with mean or rate *λ*
*T*(*n*, **g**)	Tubbs's model for *n* input electrons and gain **g**, see [11]
*λ*	parameter of the Poisson distribution, exclusively

The probability density function of a distribution is denoted with the symbol of the distribution followed in brackets by the variable and the parameters separated by a semicolon, e.g. 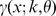 for the gamma distribution.

Each photon that hits the detector may cause the emission of a photoelectron. The probability of this event is the quantum efficiency, 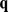, which depends on the detector material and the wavelength of the light. The probability of obtaining 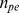 photoelectrons from 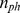 photons is given by the binomial distribution 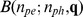. The probability of getting 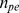 photoelectrons from a light source is hence given by a combination of mutually exclusive events of joint probabilities of the Poisson distribution and the binomial distribution. This is again Poisson distribution where the mean is the product of intensity and quantum efficiency:
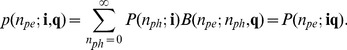
(9)


The emission of thermal and clock induced charge is also governed by a Poisson distribution with the emission rate, 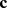. This contribution can be further decomposed into its dark current and CIC components, 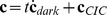, where 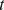 is the exposure time. An electron that enters the EM register is either a photoelectron or spurious charge. Therefore we can describe the number of input electrons, 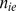, of the EM register as the convolution of the two Poisson distribution for the photoelectrons and spurious charge, respectively:
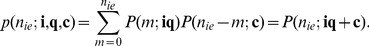
(10)


The expected number of input electrons is the parameter of the Poisson distribution: 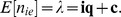 Note that 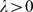 always holds, since 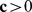, even though it might be very small. Hence we don't need to consider the case where 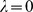.

#### The EM register model

There are two similar ways to describe an EM register with gain 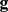. [10] suggests the gamma distribution for the probability to get 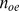 output electrons:
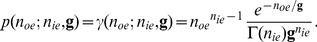
(11)


The other possibility was suggested by R. Tubbs [11]. His approach initially looks at two models, one of which is a cascade of Poisson processes and the other a cascade of Bernoulli trials. This goes along with our understanding of the impact ionisation during the charge transfer in the EM register. However, Tubbs finds that the difference between the models is minor and concludes that discretisation of the signal dominates the signal to noise performance rather than the internal properties of the individual gain stages. He derives the following approximation for the probability distribution, 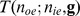, for the number of output electrons, 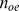, given the number of input electrons and depending on the gain factor, 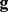:
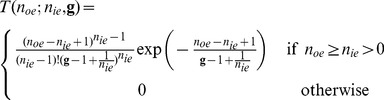
(12)


This approximation is valid for large gains and large numbers of input electrons.

We have analysed Tubbs's model. If we define
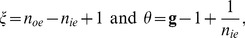
(13)then we see that eqn. (12) is a gamma distribution for 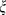 with shape parameter 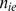 and scale parameter 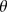,



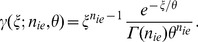
(14)Note that the scale parameter lies between the gain and gain minus one, 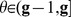. The variable 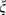 is the number of electrons created in the EM register plus one.

Using the standard result for the gamma distribution we obtain for both models the same expectation value for 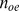,
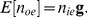
(15)


For the Tubbs models it follows from
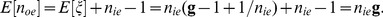
(16)


Both models fulfil our expectation that 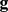 is the gain. Similarly the variance is
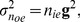
(17)in both cases. While the parameter transformation 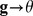 is obviously not very significant, the transformation 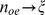 does seem to have a higher impact. However a simple rearrangement shows that 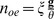. That means for large gains both models are from a practical point of view identical. Moreover, both models are already approximations of convolutions of the individual EM stages.

#### EM register simulations

**Figure 4:**
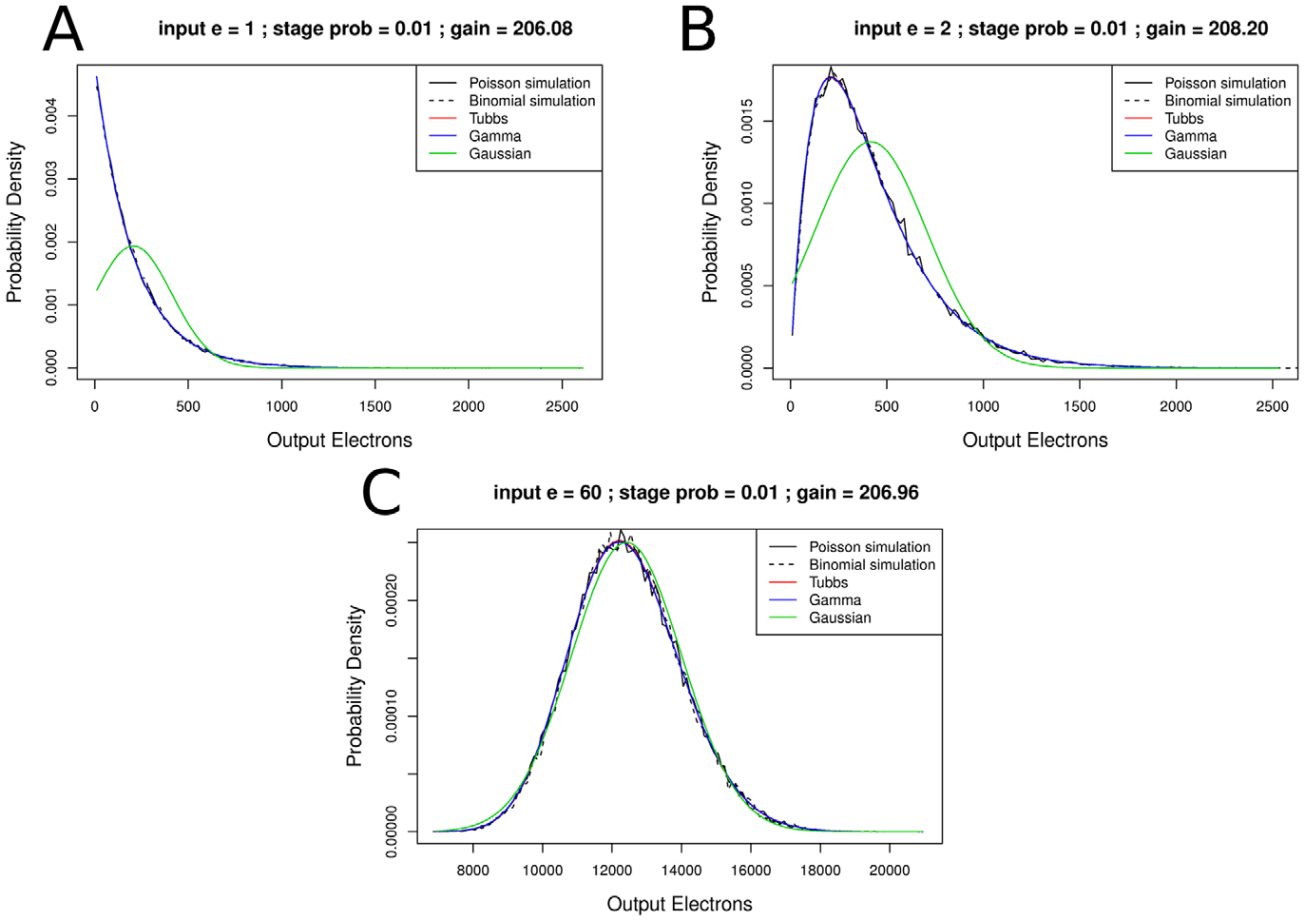
Simulation of the EM register composed of 536 stages with the Tubbs model, gamma distribution and normal distributions fitted. The parameters for the distributions were calculated from the Poisson distribution (50,000 samples). Gain is estimated as sample mean divided by the number of input electrons, the parameters for the normal distribution are sample mean and sample standard deviation. (A) 1 input electron, (B) 2 input electrons, (C) 60 input electrons. The probability to create a new electron for each existing electron per multiplication stage is 1%. That yielded overall gains between 206 and 208. Both simulations, the Tubbs model and the gamma distribution are very similar in all cases. For a high number of input electrons, the similarity of the normal distribution to the simulated data is also high. However, EMCCDs are used to measure low intensities or single photons.

To decide whether to use the Tubbs approach or the gamma distribution as a model for the EM register, we simulated the EM register, which is composed of some hundred gain stages, by modelling an individual stage with either a Poisson distribution with parameter 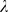 or a binomial distribution with probability 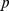 (see materials and methods). The choice of 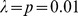 yielded the same overall gain of just above 200 for both models. This is a recommended value for EMCCDs in intensity measurement experiments, since it reduces the effective readout noise to less than one photon. We used this gain value to calculate the probability density with Tubbs's function and the gamma distribution. Fig. 4 shows the results for one, two and sixty input electrons. We calculated the gain parameter for Tubbs's model and the gamma distribution as the mean of the samples of the Poisson based simulation divided by the number of input electrons. The parameters for the normal distribution are the sample mean and the sample standard deviation of the same simulation. We see a high similarity between the Poisson and Binomial simulations, which is due to the low probability for an individual electron to be released. Both, the Tubbs model and the gamma distribution fit the simulation very well. From a practical point of view the distributions are undistinguishable for high gain values. The normal distribution fits the data very poorly for low numbers of input electrons. The fit improves for more input electrons. However, the higher the signal the less appropriate it becomes to use an EMCCD. The normal distribution is therefore not an appropriate model for the EM register.

**Figure 5:**
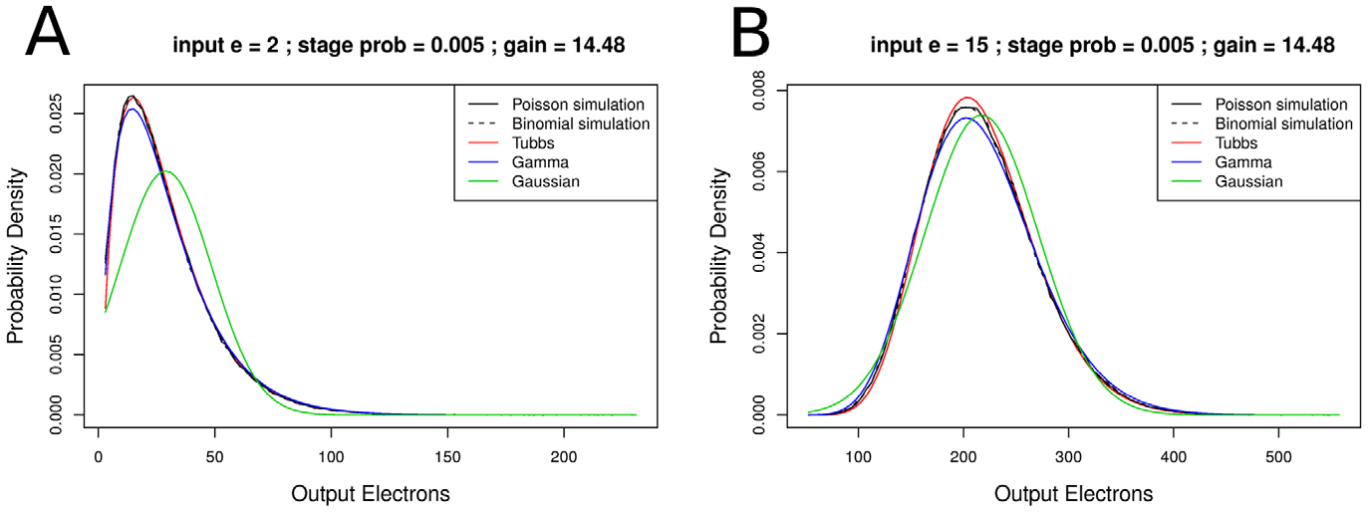
Difference of the Tubbs model and the gamma distribution for low gain settings. The EM register models are fitted to simulated data (A) 2 input electrons, (B) 15 input electrons. The probability to create a new electron per existing electron in a multiplication bin is 0.5%. That yielded an overall gain of 14.48. Sample number 250,000. For two input electrons the Tubbs model fits the data slightly better than the gamma distribution. However, EMCCDs are usually operated with much higher EM gain values.

For low gain settings, there is a clear difference between the Tubbs model and the gamma distribution as Fig. 5 shows. For 15 input electrons both models fit the simulation reasonably well. The Tubbs model is slightly sharper than the simulation whereas the gamma distribution is slightly broader, though in both cases the difference is small. For 2 input electrons the Tubbs model seems to fit the simulation better than the gamma distribution. Since EMCCDs are not operated in low gain modes, this difference has no practical consequence. Note again that both simulations are very similar.

Following the theoretical considerations and the results of the simulations, we decided to use the gamma distribution as the model for the EM register, given that it is slightly simpler.

#### The combined Poisson-Gamma distribution

The probability that 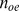 electrons leave the EM register if light with a mean intensity of 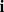 photons hits the detector is
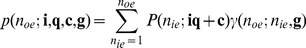
(18)

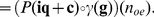
(19)


A composition of the Poisson distribution and the gamma distribution as a model for EMCCDs has also been given by [10], although quantum efficiency and spurious charge were not integrated into the model. Since we want to formulate the probability density for the whole process we have to take into account the possibility that no electron enters the EM register. Neither the gamma distribution nor Tubbs's model allows this possibility. We need therefore to expand the model for the EM register such that
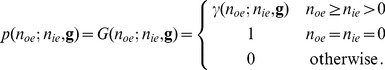
(20)


This means that we assume that the EM register does not produce any electrons if the input is zero and that all spurious charge is created before the EM register, which is not the case in reality. [13] considers this point, while others (e.g. [15]) make the same assumption that we do here. We obtain with 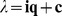 (i.e. 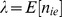) the composition.
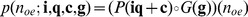
(21)

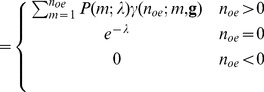
(22)


We assume 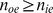, i.e. that at least as many electrons are leaving the EM register as were entering the EM register. Therefore the sum in eqn. (22) runs to 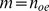.

**Figure 6:**
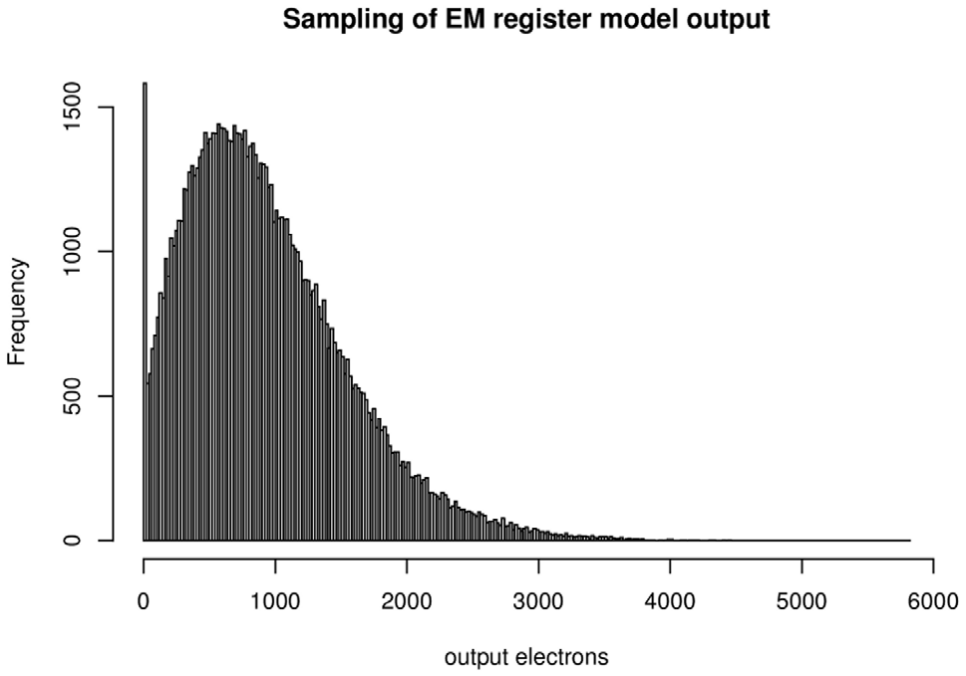
Sampling of the distribution of output electrons of an EM register for 5 photons. The simulation shows the effect of low light intensities in the model. A large number of bins is chosen to emphasise the spike at 0 output electrons. The spike is the result of the assumption that zero input electrons will always yield zero output electrons. Other parameters: 90% quantum efficiency and 0.02 electrons spurious charge per pixel. Sample number 100,000.

The additional 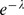 term is insignificant if the number of photons is large, say if 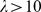, but it affects the distribution for small 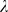 as illustrated in Fig. 6. The sampling was done as for Figs. 4 and 5, except that the number of input electrons, 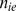, was sampled from (10).

However, eqn. (22) is rather unwieldy. It also appears to be easier to consider the series
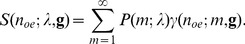
(23)rather than the finite sum of eqn. (22). For high gain, the difference between the equations is negligible. Numerical estimates yielded
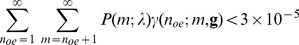
(24)for any 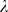 and 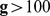, whereas the expression is largest for small 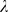. A rearrangement of terms in eqn. (23), recalling that 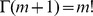, and the substitution 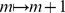 leads to



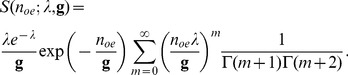
(25)The sum of this series is known to be.
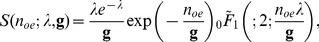
(26)where 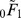 is the regularised hypergeometric function. We use an identity to transform the hypergeometric function into a modified Bessel function of the first kind:



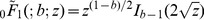
(27)Further rearrangement leads to
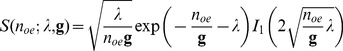
(28)


We can write the last expression as
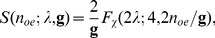
(29)where 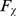 is the non-central 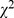 distribution for 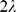 with 4 degrees of freedom and the noncentrality parameter 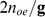.

The eqn. (22) can therefore be written as (again 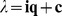):
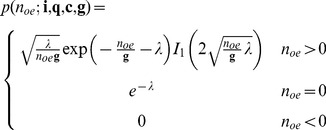
(30)


We get the following results for the expected values of 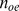 and 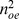


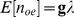
(31)

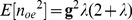
(32)


Hence the variance is given by
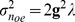
(33)


The model thus explains the excess noise factor of the EMCCD which has been measured as 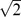 (e.g. [12]):
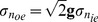
(34)


This factor is also cited by [7] and [19] who refer to [12] and EMCCD manufacturer's documentation [20], [21].

#### Including the readout noise

The last component of the model is the readout noise which is modelled by a normal distribution with standard deviation 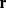, 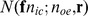. The readout register converts the analogue signal into discrete image values. The analogue-to-digital proportionality factor, 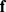 (A/D factor, sometimes referred to as amplifier sensitivity), is the number of electrons per image value. In other words, we have 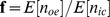. Therefore the probability of measuring image value 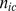 in a pixel for a given set of EMCCD specifications 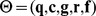 and 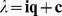 can be written as:
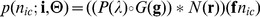
(35)

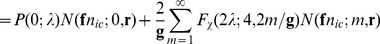
(36)


If we apply the approximation given in [13] we obtain
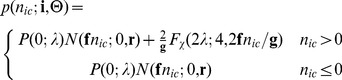
(37)

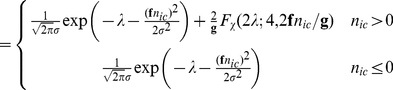
(38)


This is the PGN noise model likelihood of the EMCCD in its general form. These equations appear in a similar form in [19] and [15] using eqn. (28). The case distinction is necessary since the second summand is undetermined for 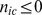. However such values are likely to appear in low-light imaging.

### Estimation of the Parameters

To use the model, we need to estimate the quantum efficiency, 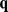, the spurious charge, 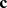, the EM gain, 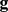, the readout noise, 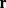, and the A/D factor, 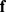.

#### The analogue-to-digital proportionality factor

To estimate the A/D factor, or sensitivity, 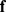, we apply the mean-variance test [22] to a series of image stacks, each stack taken with a different constant intensity of source light. To make sure that the probability of the image counts, 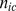, is dominated by the Poisson component, the EM gain is set to the lowest value or turned off (depending on the make of the EMCCD). Hence we have approximately 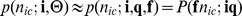. The probability for 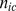 is therefore a scaled Poisson distribution with mean 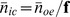 and variance 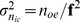. We can therefore estimate
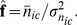
(39)


**Figure 7:**
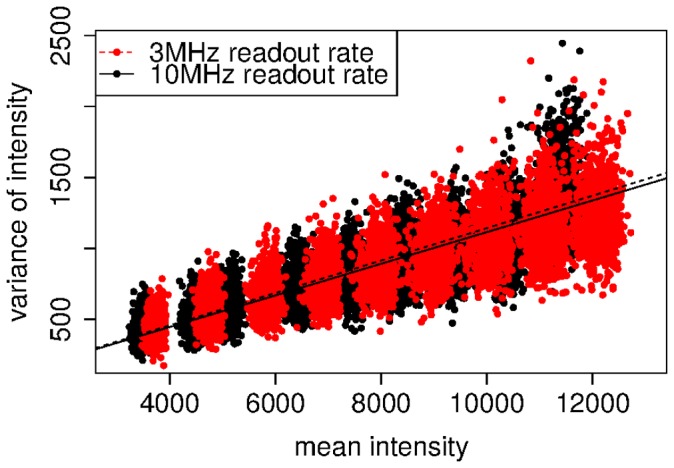
Mean-variance plot for the A/D factor estimation. Each dot represents mean and variance of the intensity of an individual pixel for 60 frames across a single data set. The values of 9 data sets are shown which appear as “blobs” in the image. Each data set was taken with a different but constant light intensity. The data shown in red was taken with 3 MHz readout rate and the data shown in black was taken with 10 MHz.

In practice, the A/D factor is estimated as the inverse of the gradient of the least-squares straight line fit to the mean-variance data, combining results from multiple intensities. Fig. 7 shows two example plots for such an estimation. The data points form a chain of overlapping “blobs” where each blob represents an individual data set with a particular light intensity. The data was taken with different readout rates. The difference of the readout rates is small, which is in agreement with the manufacturer's specification.

#### EM gain

For large gain factors, the readout noise becomes negligible compared to the EM noise. The variance of the output electrons of the EM register is according to eqn. (34) given by 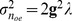. Considering the expectation for 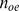, 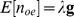 and the relations of expected value and variance between output electrons and image counts, 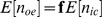 and 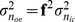, we can estimate the gain 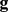 by
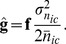
(40)


Hence, we can acquire 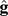 through a mean-variance test of stacks of white light images with different intensities, fitting a straight line and using the gradient. For a single intensity dataset we can estimate 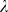 with
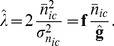
(41)


**Figure 8:**
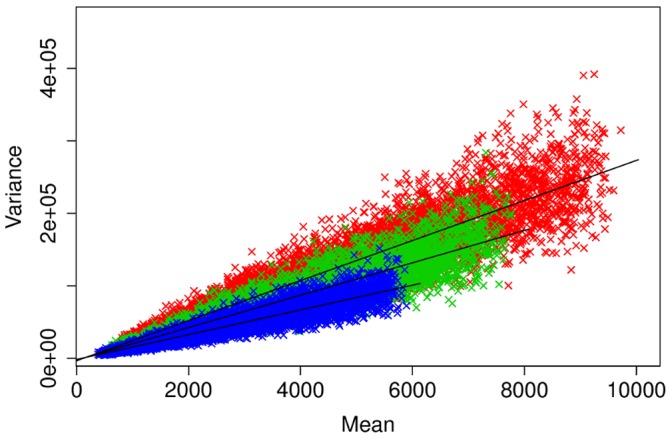
Mean-variance plot for the EM gain estimation. Mean and variance were calculated from 60 values per pixel. Shown are data sets taken with an EM gain setting of 150 (blue), 200 (green) and 250 (red). The black lines indicate the fitted linear model. Each data set contains 932970 points; 7500 randomly sampled data points are shown.

[23] (supplement) suggest to estimate the gain by manually fitting eqn. (36) to stacks of white-light images. This approach was also adopted by [19]. Fig. 8 shows an example plot for such an estimation. The plot of the data is club-shaped. The plot shows the data taken with three different gain settings in three different colours. The gradient of the linear model fitted incrases with increasing gain-setting.

#### Spurious charge and readout noise

We estimate the spurious charge and the readout noise from dark images. The probability distribution of a dark image for parameters 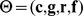 is given by
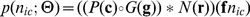
(42)


An example histogram for high gain is shown in Fig. 2. The peak around 80 image counts marks the bias offset and its width is determined by the readout noise. Nonetheless the peak is not a pure Gaussian since it also contains spurious charge. We have seen in Fig. 4A that, for one input electron, a small number of output electrons is most likely. The fat tail is caused by the amplified spurious charge. The time-dependent thermally induced component of the spurious charge is very low. In a test exposure over 3 hours and with a −80°C detector temperature, we measured 0.0023 electrons per pixel per second. This means that the spurious charge is mainly clock induced.

We use the parameters estimated from light images to estimate the remaining two parameters. We take a series of dark images and create a histogram 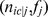 with 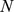 bins, where 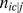 is the mean count of bin 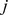 and 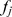 is the frequency of occurrence. We estimate the readout noise and spurious charge from the maximum of the log-likelihood.
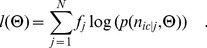
(43)


The probability 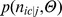 is the model for the dark image eqn. (42) with 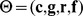. The parameters 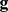 and 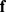 are the fixed estimates we obtained earlier. That means we estimate.
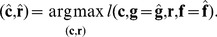
(44)


**Figure 9:**
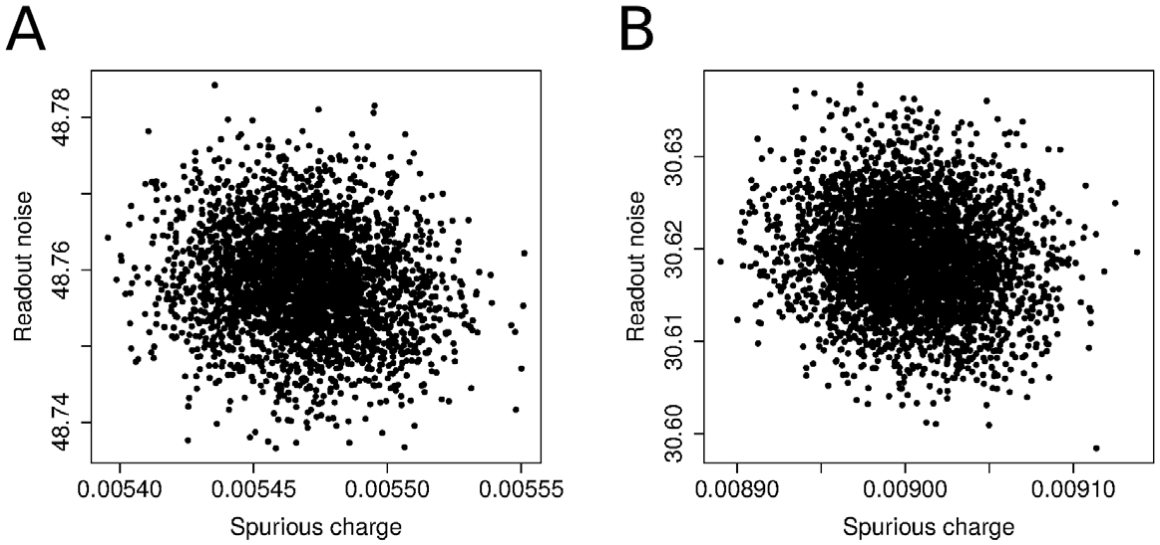
Maximum likelihood estimation for spurious charge (electrons/pixel) and readout noise (electrons). Shown are the samples of the likelihood function (see Material and Methods) of readout noise and spurious charge for a set of dark images. The images for (A) and (B) were taken with 3 MHz and 10 MHz readout rates respectively. The manufacturer gives ca. 53 electrons readout noise for the settings used for (A) and ca. 32 electrons readout noise for the settings used for (B).

Fig. 9 shows the results yielded by a distribution sampler (see Materials and Methods) for eqn. (44). The data was taken with 10 MHz and 3 MHz readout rate. The manufacturer gives the readout noise as 53 electrons and 32 electrons respectively.

#### Quantum efficiency

It is difficult to measure the quantum efficiency. In particular it is difficult to know how many photons actually hit the detector. We therefore take the quantum efficiency from the manufacturer's specification.

### Validation

**Figure 10:**
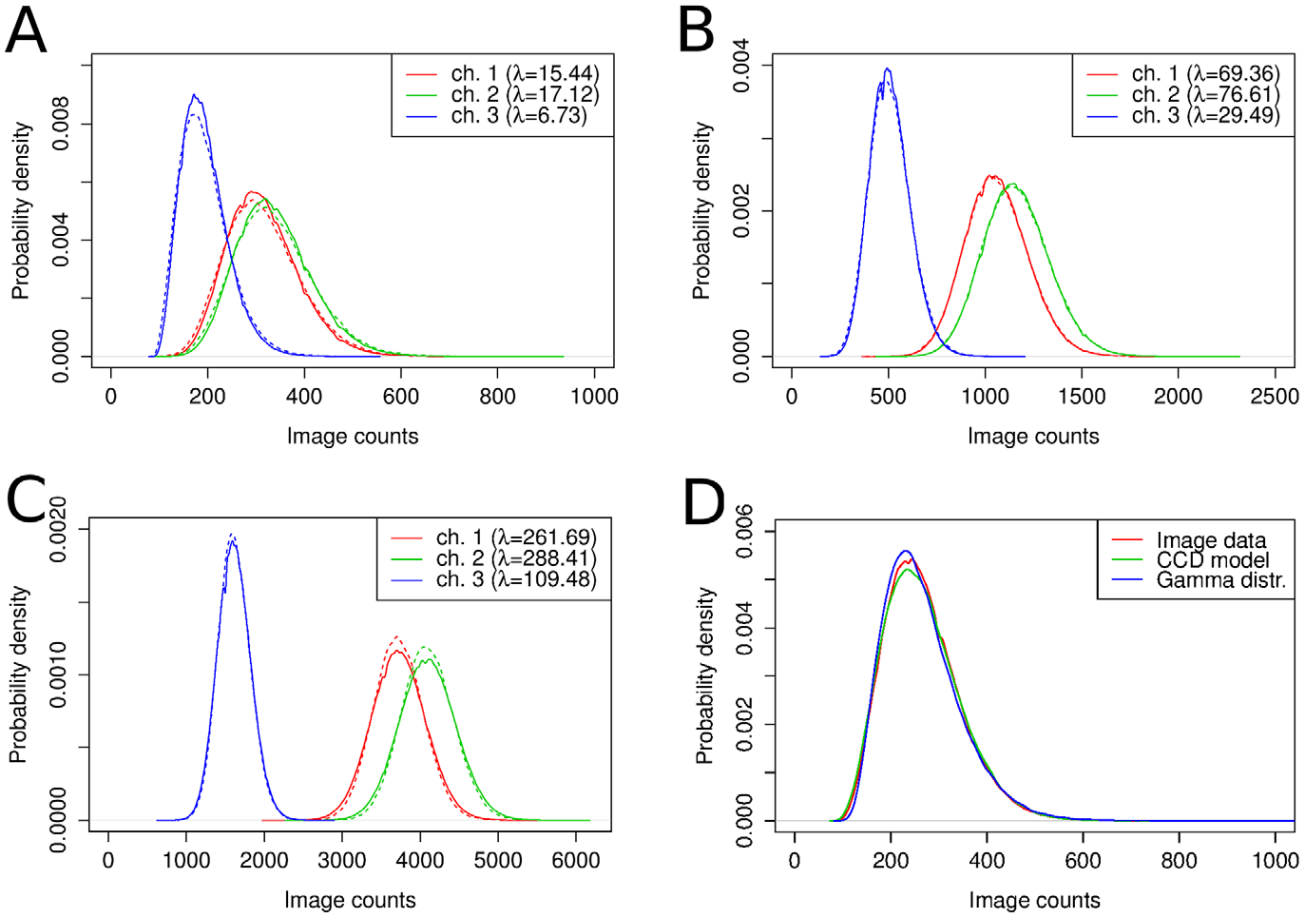
Comparison of the density of stacks of white light images with the model density. Each panel shows intensity density of a data set of 100 frames and 3 optical channels (continuous lines). The data sets were taken with different intensities; (A) low intensity, (B) medium intensity and (C) high intensity. The model densities are drawn with dashed lines. The common gain estimate is 175.9. The difference in intensities for different channels of the same data set is caused by the splitter optics. Panel (D) shows the comparison of the density of the image counts with the density of the CCD model and the density of a convolution of a gamma distribution with a normal distribution. The shape parameter of the gamma distribution is half the number of input electrons of the EM register, while the scale parameter is twice the estimated EM gain. The estimated number of average input electrons is 8.7. For higher light intensities, the densities become more similar.

To see if the model is a good description of a real EMCCD, we compared the model with the intensity density of a white light images with a short exposure time and a dim light source. The data sets were taken through an Optosplit III image splitter (Cairn Research) as we use it for single molecule imaging [24]. This Optosplit divides the image into three spectrally distinct but spatially identical channels. We took image series of 100 frames with 3 different light intensities. The intensity density for each data set and each channel is shown in figure 10 (continuous lines). We estimated the gain according to eqn. (40) using all data sets. The expected number of input electrons was estimated according to eqn. (41) for each channel and each data set separately. The input electrons are photo electrons and spurious charge. The result is shown in figure 10. The output is given in image counts with an A/D factor of 12.7 electrons per image count (taken from the manufacturer's performance sheet). The density functions of the model closely resemble the densities calculated from the image data.

Panel (D) of Fig. 10 shows the comparison of the density of image intensities, the model density and the density of a combined gamma-Gaussian distribution. The shape parameter of the gamma distribution is half the number of input electrons of the EM register and the scale parameter is twice the EM gain. The normal distribution is parametrised with the estimate for the readout noise in all cases. For higher numbers of input electrons the model and the gamma distribution become more similar so that the gamma distribution can be used as a simplification. It is important to remark that simply applying the gamma distribution from the start does not tell us anything about the meaning of the parameters. We would also lose the understanding about the assumptions and approximations that have been made and therefore the limitations of the model.

## Discussion

We have introduced a stochastic model for EMCCDs. This model does not just give expected value and error as descriptive parameters, but provides a full probability distribution. The model agrees with that in [15] and theoretically confirms the excess noise factor of 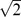 that was found empirically by other researchers [12]. Understanding noise model properties in detail allows acceptable approximations to be made where necessary for individual problems.

The parameters of the model can change for different settings of readout rate, pre-amplification gain, EM gain, CCD temperature and vertical clock speed. The model parameters may also be different for other modes of operation, such as binning or frame transfer mode Therefore we recommend to estimate the model parameters for each set of settings. We recommend the following procedure:

1. *Bias offset correction*. For each experiment a number (50–100) of dark images is taken with the same settings as the images that are to be corrected. The bias is removed according to eqn. (5). The row, column and total means can hereby taken over a range of images.
2. *Sensitivity estimation*. A number of white light images series should be taken, each with EM gain minimal or if possible with EM gain off, using a constant light source. We put water between the light source and the CCD to make the light more homogeneous. Each series needs to contain enough images to calculate the intensity variance on a pixel by pixel basis. The image counts should be different for each series, either by changing the intensity of the light source or by changing the exposure time between each series. The series should cover a wide range of image counts, but pixel saturation must be avoided. For each pixel of each series, the mean, 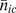, and variance, 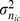, of image counts should be determined and a straight line fitted to the data from all pixels and image series to give the sensitivity using 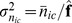. Sensitivity estimation need only ever be performed once for a particular EMCCD and combination of settings.
3. *EM gain estimation*. This can be performed in a similar fashion to the sensitivity, by fitting a straight line to the pixel mean-variance data from a series of constant-intensity image stacks each of different intensity, except that the EM gain should be set to the value for which the gain needs to be measured, i.e. the value of EM gain used in experiments. From eqn. (40), the gradient of the mean-variance fit will give the EM gain. The EM gain estimation need only be performed for each individual EMCCD and settings occasionally, but should be periodically repeated because it is known to change as an EMCCD ages, at a rate depending on how the EMCCD is used [21], [25], [26]. According to a manual of Hamamatsu [21] the ageing effect is most prominent at the beginning of the EMCCD usage. We did not explore the ageing behaviour.
4. *Spurious charge and readout noise estimation*. This can be performed from dark images according to eqn. (44). The same dark images used for the bias correction may be used. The maximum likelihood estimation is applied to a histogram of the intensities of the bias corrected dark images.

The most suitable setting for the EM gain depends on the experiment type. For low-light intensity measurements the gain needs to be high enough to overcome the readout noise. A signal of 10 electrons would disappear in a readout noise of 50 electrons. However, if the signal is multiplied by a factor of 200, the signal to readout noise ratio would be 40∶1. EMCCD manufacturers often limit the EM gain to around a factor of 1000 and some have implemented an additional setting to unlock EM gains beyond factor 300. This is done to protect the EM register from ageing. For photon count experiments however, [7] argue that the EM gain should be as high as possible. The reason for this lies in the shape of the distribution of output electrons for one input electron, see Fig. 4A. The mode of the distribution is at one output electron. That means even for a gain of 200, the likelihood to get less than 50 output electrons from one input electron is very high. The situation immediately changes for two input electrons, where the mode of the probability density is much higher. Consequently, photon counting experiments, where the aim is to detect single photons need to be treated differently from intensity measurements, where the number of photons in a pixel is estimated.

Although we have considered a wide range of influences on the detected image counts, not all of the factors need to be considered all the time. Thanks to the EM register the readout noise is very low compared to other sources of noise. In some cases it can be ignored, which simplifies eqn. (36) to a form of eqn. (30) that uses the relationship 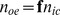. The dark current is very low if the detector is cooled. For short exposure times, it might even be ignored. We took a series of dark images with a 3 hour exposure time and estimated a dark current of 0.0023 electrons per pixel and second or 8.23 electrons per pixel and hour. Since the clock induced charge is a time constant offset, the dark current can be more precisely estimated by fitting a linear model to dark image data collected with different exposure times.

The CIC causes rare single pixel events. Individual events can have moderately high image counts. It is one advantage of the model presented here that it appropriately takes such events into account. This can for instance affect feature detection methods that compare the probability that there is a feature at a particular location to the probability that there is only background and noise.

Conventional CCDs have a lower readout noise than EMCCDs. That means EMCCDs lose their advantage over conventional CCDs if the expected number of photons is high, see e.g. [27]. At relatively high temperatures the contribution of the dark current increases significantly. Since the number of photo-electrons is low, the dark current would considerably contribute to the image. Therefore, EMCCDs normally are operated at low temperatures. We made the assumption that no spurious charge – neither clock induced charge nor dark current – is created in the EM register. As far as this assumption is valid, the model would be unaffected by higher temperature, since it only would affect the number of input electrons. However, in reality spurious charge also is created in the EM register [13]. For the specification range that we considered, our assumption is justified.

The temperature of the detector chip also affects the EM gain. We did not measure the temperature dependency of the EM gain. Technical notes of the chip manufacturer e2v [28] and CCD manufactures suggest that the EM gain smoothly increases with decreasing temperature. The chance to create an extra electron in an EM register bin is very small. This is true for any temperature and causes a high similarity for the underlying bin models, the binomial distribution and the Poisson distribution. Taking into account the smooth dependency, we do not expect the temperature to change the principal way of functioning of the EM register, but only to affect the values of the parameters. We would therefore expect our model for the EM register to be valid for any temperature. However anyone using the model should confirm this when determining parameters for their detector setup, taking extra care if operating outside the manufacturer's recommended regime.

The distribution of image counts that result from a constant light source of low intensity is not normal, as the examples in figures 10 and 4 show. Under such circumstances the descriptive parameters mean and standard deviation have questionable value and to give intensity measurements as mean plus-minus standard deviation can be misleading. The remarks about the gain setting for photon counting experiments emphasize this. Even though the expected number of image values per photon lies well above the readout noise, many photons will yield much fewer image counts and be concealed by the noise.

## Materials and Methods

The images were taken with an Andor iXon+ EMCCD with a CCD97 detector chip from e2v [28] using 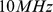 readout rate, 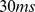 exposure time and 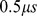 vertical clock speed at 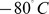 sensor temperature. Simulations were done using R. Matlab was used to aid the derivation of the equations. The parameter estimation was implemented in C++. The optimisation of the maximum likelihood was done using MultiNest [29].

We simulated the EM register with 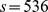 stages (the number of stages of the CCD97 chip) with two models. In the first model we assumed that the number of electrons released in one stage by each incoming electron is governed by a Poisson distribution, 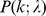, where 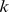 is the number of created electrons and 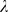 the emission rate or expected number of electrons created by a single incoming electron. For 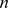 incoming electrons we have to calculate the convolution of 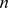 Poisson distributions with the same emission rate, which is simply a Poisson distribution where the emission rate is multiplied by the number of electrons, i.e. we model each stage with 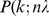). The second model assumes that each incoming electron can release one electron by impact ionisation, thus that the number of emitted electrons in a single stage is governed by a binomial distribution, 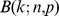 where 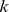 is the number of new electrons, 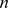 the number of incoming electrons and 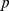 the probability of impact ionisation. The probability density function for the simulation of the EM register can be expressed as
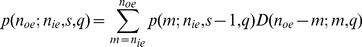
(45)

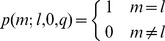
(46)where 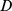 is either the binomial distribution, 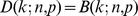, or the Poisson distribution 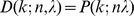.
